# The Clinical Encounter as a Gateway to One Health: Rethinking Communication Education in Veterinary Curricula

**DOI:** 10.3390/ani16213365

**Published:** 2026-10-25

**Authors:** Rita Payan-Carreira, Margarida Simões

**Affiliations:** Comprehensive Health Research Centre (CHRC), Departamento Medicina Veterinária, Escola de Ciências e Tecnologia, Universidade de Évora, 7004-516 Évora, Portugal; mpsimoes@uevora.pt

**Keywords:** veterinary education, communication skills, One Health, competency-based veterinary education, risk communication, science communication, interprofessional education, veterinary curriculum, public health communication, client communication

## Abstract

Communication is an accepted clinical competency in veterinary medicine, taught and increasingly assessed during the veterinarian–client consultation. However, the same encounter is also the profession’s most frequent point of contact with antimicrobial stewardship, zoonotic risk, food safety and public trust in science, and the population- and public-facing communication demanded by the One Health role remains comparatively overlooked in veterinary curricula. This narrative review reframes the clinical consultation not as the boundary of veterinary communication but as its gateway. Synthesizing current literature on communication teaching in veterinary education, it argues that provision is heavily weighted towards the dyadic core and comparatively silent on interprofessional, community, public health and digital communication. On this basis, it proposes an integrated Communication Competency Continuum, in which five transversal competencies (relational, informational, behavior change, risk and audience adaptation) are progressively re-contextualized from the clinical dyad outward to the One Health mandate, anchored in established competency frameworks and made explicitly assessable. Offered as a conceptual contribution rather than a prescription, the continuum is not intended to replace clinical communication training, which should be preserved, but to extend it, so that veterinary communication education matches the breadth of the profession’s role.

## Simple Summary

Veterinarians spend much of their working lives communicating, not only with the owners of the animals in their care, but also with farmers, colleagues in other professions, public health authorities, the media and the wider public. Veterinary training has become very effective at teaching communication for the consulting room, but it pays far less attention to these wider audiences, even though communicating clearly about issues such as antibiotic resistance, disease outbreaks and food safety is central to the profession’s role in protecting animal, human and environmental health together, a concept known as One Health. This article argues that the skills learned in the consulting room are the starting point for all these broader conversations, and proposes a practical framework to help veterinary schools extend communication teaching outward, step by step, from the individual animal owner to the whole community and population.

## 1. Introduction

From the Latin *communicare*, “to make common,” “to share” or “to relate”, communication is less about transmitting information than about building shared understanding [1]. In veterinary practice, this shared ground must be built with clients who speak on behalf of patients who cannot, making the relational dimension of communication particularly critical. Communication is now widely recognized as a core clinical competency in veterinary medicine rather than being regarded an isolated “soft skill” to be learned by example. Over the past two decades, the profession has adapted validated models from human medicine, most prominently the Calgary–Cambridge guide, a structured model of the consultation that divides the encounter into defined tasks (initiating the session, gathering information, physical examination, explanation and planning, and closing) and the communication skills that serve them, to the structure of the veterinary consultation [2], and has embedded communication as an explicit outcome in competency-based curricula [3,4], reflecting a sustained pedagogical interest in education and communication in veterinary clinical practice [5]. Although communication teaching in veterinary education is increasingly structured and experiential, that integration remains uneven and often concentrated on clinical encounters [6]. The rationale is well evidenced through the quality of the veterinarian–client exchange that shapes the spectrum of care and shared decision making [7,8], the client satisfaction and adherence [8], the reduction of incidence of complaints [9], the improvement of communication among the veterinarian team [10], and even the enhancement of practitioners’ wellbeing, with relational demands identified as a driver of burnout [11,12]. In line with this mindset, communication training has been gradually included in veterinary curricula or offered as supplementary training. Reflecting the importance of communication competences in healthcare contexts, international accreditation of Veterinary Medicine programs requires, in many countries, the universities to overtly teach and assess communication skills, with a particular focus on clinical communication [4,13,14,15]. Communication is part of how the decision is constructed, negotiated and implemented. Literature related to the European Association of Establishments for Veterinary Education (EAEVE) already identifies communication as embedded in several Day One competences, rather than as an isolated subject. Targeted training measurably improves client-centered communication in practice [16] with dedicated pedagogies, such as simulated clients, objective structured assessments and structured teaching of emotionally demanding conversations such as euthanasia [11,17,18,19]. The dyadic core of veterinary communication is therefore real, teachable and, in a growing number of schools, trained and assessed.

However, the same clinical encounter that those models describe so well is rarely “only” a clinical encounter. When a veterinarian justifies or declines an antimicrobial prescription, counsels an owner on a zoonosis, advises a breeder on biosecurity or reassures a client about a vaccine, the consultation becomes an act of public health and One Health communication conducted at the consulting room table. Recent evidence makes this explicit: companion-animal veterinarians raise One Health topics in most routine appointments [20], and the clinician has been described as a first line “One Health messenger” during everyday practice [21]. Therefore, communication should no longer be taught primarily as a consultation technique, but as an integrated professional competence underpinning critical thinking, decision making, leadership, collaboration, public engagement and One Health implementation [22]. This is precisely the insight captured, almost a decade ago, in the concept of “One Communication”—communication as the operational link that allows veterinary medicine to deliver One Health beyond the individual patient [23]. The clinical consultation, in other words, is not the ceiling of veterinary communication but its entry point as communication is the enabling mechanism connecting the other One Health building blocks: collaboration, coordination and capacity building [24].

The main problem/challenge is that curricula tend to treat this gateway as an endpoint. Communication is taught, modeled and assessed as a competency that begins and ends in the veterinarian–client dyad, while the population- and public-facing dimensions of the role remain comparatively overlooked. The consequences are visible from the client side: German owners of both companion and exotic animals report receiving less information on zoonoses and antimicrobial resistance than they would want from their veterinarians [25,26]. They are also visible at the level of the profession’s expanding roles. The COVID-19 pandemic underscored both the value and the under-use of veterinary public health expertise and prompted calls for more visible veterinary involvement and leadership within One Health [27,28,29]. Contemporary challenges, such as antimicrobial resistance, emerging and re-emerging zoonoses such as highly pathogenic avian influenza [30], are reshaping the veterinary remit in an increasingly globalized world [31]. They position the veterinarian as an interprofessional collaborator [32], in health emergencies whose contribution nonetheless remains under-recognized [29,33], and as an educator of communities [34]. Each of these represents, at its core, a communication task, and each demands competencies that the dyadic consultation does not build by itself [35]: addressing audiences with whom one has no prior relationship, communicating one-to-many and through the media, and framing messages for behavior change at population scale [36]. Globally, veterinarians engage in systems practice, using both professional and societal communication for better team communication, interprofessional collaboration, public and risk communication, leadership, advocacy and responsible use of AI tools, which are increasingly integral to how veterinary information is produced, delivered and shared with clients and the public [32].

These demands are intensifying in a digital and contested information environment. Health misinformation now extends to animals: canine vaccine hesitancy has been shown to spill over from human anti-vaccine sentiment [37], even if it still represents a minority [38], which brings measurable policy consequences. In contrast, evidence suggests that online corrections have a weak–moderate effectiveness [39]. Veterinarians are increasingly present on social media [40] and are being urged to counter animal health misinformation [41]. Doing so effectively requires them to adopt, within the veterinary domain, principles developed in the broader science communication field: a shift from a “deficit” model of one-way information transfer toward dialogic, engagement-oriented communication [35] and sustained attention to preserving public trust in expertise [42]. Despite this need to go beyond communication in clinical consultation settings, little emphasis is transposed to most veterinary curricula.

There is, however, no need to start from scratch. A recent scoping review of communication training in veterinary education suggests a growing but still uneven provision, oriented largely toward the clinical consultation [6]; and established competency frameworks already provide anchoring points for a broader conception: the Competency-Based Veterinary Education (CBVE) framework, in which communication is a defined competency domain, and its entrustable professional activities [4,43], the European Day One Competences [44], the “Communicator” role of CanMEDS (originally the Canadian Medical Education Directives for Specialists) [45], and articulated One Health core competencies [46,47]. What is missing is an integrative account that connects these strands along a single developmental line. Heise and colleagues [48] encourage veterinary medicine education institutions to incorporate interprofessional education and collaborate with other institutions of various professions to boost interprofessional communication. According to their pilot study results, the blended interprofessional training approach showed promising results, highly needed in current dynamic workplaces.

This article advances this argument. Framing the clinical encounter as a gateway rather than a boundary, it synthesizes the current literature on communication in veterinary education, shows that it is heavily weighted towards the dyadic core and comparatively silent on interprofessional, community, public health and digital communication [49], and propose an integrated Communication Competency Continuum that extends the established clinical core outward to the One Health mandate, anchors each stage in existing competency frameworks and specifies how the resulting competencies can be taught and assessed. As a narrative rather than systematic review, it draws on a selective, illustrative body of evidence, and the framework it advances is offered as a conceptual contribution to be tested and refined rather than as a definitive prescription.

## 2. Materials and Methods

This narrative review is based on a purposive, non-systematic synthesis of the literature. Relevant sources were identified through searches of Web of Science, Scopus and PubMed/MEDLINE, complemented by hand-searching of the Journal of Veterinary Medical Education and by backward and forward citation tracking of key papers. Search terms combined veterinary education and training with communication and its wider contexts (e.g., “veterinary”/“veterinarian” AND “communication”/“communication skills”/“curriculum” AND “interprofessional”/“One Health”/“public health”/“risk communication”/“science communication”/“community”). Publications from 2020–2026 were prioritized, with earlier fundamental works retained where relevant; only English-language, peer-reviewed articles and credible framework documents were considered. As with a narrative review, sources were selected illustratively to develop and support the conceptual argument, not through a systematic or reproducible protocol; thus, the review does not claim exhaustive coverage.

## 3. Core Principles of Clinical Communication in Veterinary Practice

Over the last two decades, clinical communication has moved from being treated as an innate attribute of the “good vet” to being recognized as a defined, teachable and assessable clinical competency. Central to this shift has been the adaptation to veterinary practice of consultation models developed in human medicine, most influentially the Calgary–Cambridge guide, which was mapped onto the veterinarian–client encounter, across companion-animal, equine and production-animal practice alike, and articulated in dedicated veterinary texts [2,50]. Communication is now embedded as an explicit outcome in competency-based curricula and their assessment architecture, appearing as a distinct competency domain in the CBVE framework and among the core entrustable professional activities expected of graduating veterinarians [3,4,43]. The consultation is thus no longer left to chance; it is described, taught and, increasingly, examined.

The rationale for this investment is well supported by evidence generated at the level of the clinical encounter. The quality of the veterinarian–client exchange shapes shared decision making, but observational work using validated instruments has shown it to be frequently limited in practice and strongly conditioned by the veterinarian’s own attitudes toward the informed client [7,51]. Contemporary accounts extend this beyond a single “gold-standard” recommendation toward contextualized, spectrum-of-care conversations that foreground the client’s circumstances, values and financial constraints [52], and the field continues to examine how far such communication should pursue genuine partnership rather than persuasion in supporting client behavior change [53]. Here, behavior change communication refers to helping clients and, beyond the clinic, producers and the public, to adopt and sustain recommended practices, such as treatment adherence, responsible antimicrobial use, biosecurity and preventive care; it matters because veterinary and public health outcomes depend less on the information delivered than on whether behavior actually changes. This is well illustrated in production-animal practice, where the veterinarian’s communication style and the relational context shape whether farmers enact herd health and welfare advice [54,55].

Communication process is, in turn, linked to consultation outcomes such as client satisfaction [8], while communication breakdowns are a recurrent source of client complaints and their downstream effects on the wider practice team [9]. The relational demands of client-facing work are increasingly recognized as a contributor to practitioner burnout, tying communication competence to the profession’s wellbeing agenda [12]. Crucially, these skills are demonstrably trainable: targeted, on-site education measurably increases client-centered communication in practice [16], and a growing pedagogy, namely simulated and standardized clients, structured teaching of emotionally demanding conversations such as euthanasia and scalable digital tools among other strategies, now supports their development and assessment [11,17,18,56,57].

This research provides a solid foundation to build upon for future ideas on veterinary communication, rather than a weak argument to be dismissed. Its defining feature, however, is also its principal limitation: both the models and the evidence are, by design, dyadic. They describe, refine and measure communication within a single tripartite relationship (the veterinarian and the individual client, mediated by the individual patient) situated in the consulting room [2,50]. The competencies are framed as evolving and terminating at the individual client rather than as transferable to audiences, formats and stakes that lie beyond the consultation; indeed, surveys of practitioners have documented the veterinarian–client relationship as the reference point for communication skill, alongside a perceived gap between the training received and the demands of practice [58]. Where the curricular emphasis has been characterized at all, it remains oriented primarily toward this clinical dyad [6]. The result is a well-developed core with comparatively little attention to what surrounds it.

Nonetheless, the skills cultivated within this dyadic core are not narrowly clinical. Building rapport and demonstrating empathy, eliciting and organizing information and explaining it intelligibly, negotiating decisions and supporting behavior change, conveying risk and uncertainty, and adapting to a given interlocutor are all “transversal” communication competencies that happen to be first taught in the consultation. Concurrently, a modeling experience on cultural humility, guided debriefing and reflective journaling (students’ structured written reflection on their own encounters, used to build self-awareness and empathy), prompted the existing veterinary curriculum to a stronger opportunity to teach cultural competency, while providing for both a practice and learning standpoint for empathic professional encounter [59]. It is precisely because they are transversal that they can be extended: the same relational and explanatory capacities that a graduate applies to a worried owner are those required, though re-contextualized and with new demands attached, when addressing a farming community, a public health team or a skeptical online audience. Understood this way, the clinical core is not the boundary of veterinary communication but its point of departure. The sections that follow trace how the veterinarian’s role, and therefore the communicative demands placed upon it, extend outward from this core along a Communication Competency Continuum, which will be developed into an integrated framework in Section 6.

## 4. The Expanding Professional Role—Why the Dyad Is No Longer Enough

The veterinarian described in the previous section, a clinician in dialogue with an individual client, is only one side of a profession whose responsibilities have widened considerably. Contemporary veterinary practice spans from herd and production-animal health, food safety and inspection, laboratory and field research, industry and regulatory advice, and to a formally recognized role within public health systems [31]. Across these settings, the veterinarian acts less as the sole decision-maker for a single patient than as an expert, adviser and risk communicator serving populations, producers, colleagues and authorities. This widening scope is increasingly codified: recent work has set out the Day One public health competencies expected of every graduate, positioning One Health not as a specialist add-on but as a core component of veterinary medicine [47].

Much of this expansion is driven by the One Health agenda and the shared challenges that define it: antimicrobial resistance [60], emerging and re-emerging zoonoses such as highly pathogenic avian influenza [30,61] and pet-associated spillover [62], food safety [63] and climate-sensitive disease [64]. The COVID-19 pandemic exposed both the value and the under-used veterinary public health expertise and community interface [27,28,29,33,62]. These challenges do not merely add tasks to an existing list, but rather reshaping a professional profile toward one that is intrinsically intersectoral and public facing. These responsibilities are particularly significant for food-producing animals, which account for a large share of global antimicrobial use. In the EU, around 62% of antimicrobials are used in food-producing animals [65], and this use is directly linked to antimicrobial resistance, food safety and environmental health. Veterinarians are therefore increasingly expected to communicate publicly on responsible antimicrobial use and on the food system, not only with individual producers but also with policymakers and the wider public [66].

This shift places increasing demands on veterinarians’ public communication role, yet this is precisely the area in which their professional education often provides the least preparation. The profession occupies a distinctive position: it holds statutory public health responsibilities, from notifiable-disease reporting to food-safety inspection and sanitary authority, while retaining the everyday trust of the communities it serves, a combination that makes the veterinarian a natural front-line communicator on zoonoses, antimicrobial resistance and biosecurity, overall participating in public health safeguard [67]. However, available evidence suggests this potential is unrealized at the point of contact, and owners of both exotic and companion animals report receiving less information on zoonoses and antimicrobial resistance than they would want from their veterinarians [25,26]. The mandate also reaches beyond warning and informing, toward sustained community education and preparedness, ranging from antimicrobial-resistance awareness campaigns to disaster readiness; all activities in which veterinary teams are increasingly expected to engage [34,36]. Discharging it, moreover, requires working effectively across sectors and professions, an interface for which veterinary and medical trainees alike are underprepared [32,68].

The unifying characteristic of these roles is that each, at its core, is a communication task, and each grows out of, even though quickly expanding it, the relational and explanatory skills cultivated in the consulting room. This is the insight condensed in the notion of “One Communication”: communication is the operational link that allows veterinary medicine to translate individual clinical encounters into population and One Health benefit [23], and it is already latent in everyday practice, where One Health topics surface in most routine appointments [20]. Recognizing the expanded role therefore raises practical questions that will be tackled in the next section: what specific communicative competencies does the veterinarian need beyond the clinic or the producers/farming communities, and how do they differ from those built within the dyad. How can responsible tutorship, preventive care, herd-health advisory communication and animal welfare be improved by communication literacy competency?

## 5. Communication Beyond the Clinic—Walking the Continuum Integrative Framework

Communication should no longer be taught primarily as a consultation technique, but as an integrated professional competence underpinning critical thinking, decision making, leadership, collaboration, public engagement and One Health implementation. If clinical consultation is the starting point, the contexts that lie beyond it can be read as successive widenings of the same communicative act, like as wavefront skill propagation. Each re-contextualizes the transversal competencies built in the dyad, whether relational, informational, persuasive, risk-oriented or audience-adaptive, while attaching new demands that the consultation alone does not rehearse. Four such contexts are considered here, in ascending order of scope. Together they trace the outward path of the continuum introduced above.

Moreover, the changing literacy profiles among entering higher-education cohorts challenge veterinary curricula to explicitly support advanced reading, writing, information evaluation and evidence-based communication. Weaknesses in reading comprehension may affect the interpretation of scientific literature, the critical appraisal of evidence, understanding legislation and guidelines, the interpretation of laboratory reports, the quality of clinical documentation, how to contribute to shared decision making, the quality of risk communication, the appeal to exercise preventive actions or how the writing referral letters or reports for authorities and how to communicate uncertainty.

### 5.1. Interprofessional and Intersectoral Communication

Beyond the client relationship, the veterinarian increasingly works with other professionals (e.g., physicians, nurses, ecologists, food-safety officers and public health authorities) in the intersectoral teams through which One Health is operationalized. This is not simply the clinical skill set applied to new interlocutors; it requires a shared vocabulary across disciplines, explicit negotiation of overlapping roles and the collaborative competencies, namely interprofessional communication, roles and responsibilities, teamwork that interprofessional education frameworks are designed to develop [69], and to which veterinary education can be explicitly aligned [70]. The evidence indicates that this readiness cannot be assumed: veterinary, medical and public health trainees hold divergent understandings of One Health and of one another’s contributions [68,71], and the interface between human- and animal health institutions remains an underdeveloped site of educational effort [32,72]. Where the veterinarian is frequently the connective node linking animal, human and environmental sectors, any weakness at this interface is consequential. Notwithstanding, communication literacy is key to analytical and systems thinking process, enabling creativity and collaboration, providing shared understanding for intervention towards sustainable and healthy common goals.

### 5.2. Community Communication

A second context addresses groups rather than institutions: breeders and producer associations, owner communities, schools and the lay public. Here, communication becomes outreach and health promotion, and success depends on cultural sensitivity, attention to health literacy and the capacity to engage audiences on their own terms rather than to deliver expert pronouncements [73,74]. Veterinary teams are increasingly called upon to work at this level, for example, in building disaster preparedness within the communities they serve [34]; yet, this context remains comparatively under-researched and under-taught [75], a gap that is itself telling given how much of a veterinarian’s population impact is mediated through community trust. Empathy and active-listening practice are essential for effective social awareness raising and building veterinary-client/community trust [76].

### 5.3. Public and Population Communication

The veterinarian’s public communication mandate has two faces, which are pedagogically distinct and are best treated separately.

#### 5.3.1. Reactive and Crisis Communication

During outbreaks, zoonotic incidents and sanitary emergencies, the veterinarian must communicate under pressure, work with the media and convey risk and uncertainty when public trust is contested. This is the domain of crisis and emergency risk communication, whose integrative model sets out how message content and timing must shift across the phases of an emergency [77]. Veterinary professionals have demonstrably contributed to such communication, for instance, in national COVID-19 responses [28,29] and current threats such as highly pathogenic avian influenza place frontline practitioners at the center of outbreak information flows [30]. Although this context is continuous with the clinic rather than separate from it, as the outbreak conversation frequently begins with the clinician who sees the index case, their contribution in emergencies remains under-recognized [33]. Beyond emergencies, the veterinarian may also serve as an expert witness in animal-cruelty or welfare proceedings, provide expert commentary to the media and contribute statements that inform on best practices, the drafting of legislation, regulations and influencing policy. These represent high-stakes, public-facing communication aspects for which graduates are rarely prepared. Opportunities to simulate such events are experienced as pilot experiments in institutions that consider communication skills and animal health-related crisis a growing concern [78]. To prepare in advance for possible crisis and disasters, a joint One Health proposal advocates for distinct communication styles (oral and written), for effective community and partner engagement, providing guidelines for training and best practices [79].

#### 5.3.2. Proactive Population Education and Engagement

Distinct from crisis response is the sustained, non-emergency work of population education: prevention and health promotion campaigns, responsible-ownership and antimicrobial-stewardship messaging and community engagement aimed at behavior change over time. Evidence on population antimicrobial-resistance campaigns indicates that impact depends on deliberate design, including the use of mass media, targeted messaging and definition of clear behavioral goals, rather than on information provision alone [36]. This proactive mode is arguably where veterinarians could exert their largest continuous public health influence [74], and yet it is almost entirely absent from formal training.

### 5.4. Digital and Science Communication

The final context cuts across all the others: the digital public domain. Veterinarians are increasingly present on social media [40] and are called upon to counter animal health misinformation [40,41,58,80], a demand sharpened by phenomena such as pet vaccine hesitancy, which stemmed from human anti-vaccine sentiment with measurable policy consequences [37,81]. The evidence on correcting online misinformation is sobering, underscoring that reactive debunking is at best partially effective [39]. Communicating effectively in this context therefore means adopting, within the veterinary domain, principles developed in the broader science communication field: a move from a “deficit” model of one-way transfer toward dialogic, engagement-oriented communication [35], and sustained attention to preserving public trust in expertise [42,82].

#### From Contexts to Competencies

Read together, these four contexts make a single pedagogical point. The competencies they demand (sustaining collaboration across professional cultures, engaging communities with humility, communicating risk under uncertainty, framing messages for behavior change at scale and holding a dialogue in a contested information environment) are extensions of the relational and explanatory skills of the consultation, but they are not automatically produced by it. Good performance in the consulting room does not guarantee competence before a microphone, a multidisciplinary task force or a skeptical online audience. The question this raises is empirical: how far do veterinary curricula currently develop communication beyond the clinical dyad?

## 6. What Do Veterinary Curricula Already Cover?

Communication is now an established and, in many settings, sophisticated component of veterinary education. Longitudinal data from colleges in the United States of America show it moving over two decades from the periphery toward a recognized element of professional-skills teaching [83], and integrative review confirms its centrality to clinical practice while underscoring a persistent need for training [10,84]. A recognizable core pedagogy has consolidated around it: structured consultation models operationalized for the veterinary encounter [50] and, above all, experiential learning with simulated and standardized clients, a method that a survey of AVMA COE–accredited institutions found to be near-ubiquitous, used by the large majority of programs [17,85]. This core is being actively refined through learner-driven simulation designs [86], dedicated course structures for emotionally demanding conversations such as euthanasia [11] and emerging AI-generated standardized clients [87,88], and it increasingly rests on explicit, evidence-based principles of curriculum design, such as longitudinal, developmental and helical sequencing built around skills practice and feedback [89]. All mentioned strategies are anchored within competency-based veterinary education, in which communication figures as a defined competency domain with associated entrustable professional activities [3,43,90,91]. Communication teaching, in short, is neither marginal nor unsophisticated.

Its distribution, however, seems uneven, with its center of gravity in the clinical consultation. A recent scoping review describes a growing but heterogeneous field in which teaching and assessment are best developed for the veterinarian–client dyad, like consultation structure, history taking, empathy, shared decision making and breaking bad news [6]. The dominant methods, such as consultation guides like the Calgary–Cambridge, or simulated-client encounters, are, by construction, models of that dyad [85]. The further contexts of the continuum, like interprofessional and intersectoral communication, community engagement, public health and risk communication, and the digital and science communication demands examined above, receive comparatively little, and often incidental, attention as explicit, assessed competencies.

However, this is not to say that wider contexts are wholly absent. Exceptions exist and are instructive precisely because they show what is achievable. Beyond the consulting room, individual programs have trained students in community engagement, delivering health lessons to schoolchildren while broadening cultural understanding [92], and, through service learning in community-based practice, communicating about access-to-care barriers with underserved clients [93]. Others have addressed production-animal and herd health communication, for instance, training food-animal veterinarians in the client conversations that underpin a voluntary disease-control program [94]. A smaller number reach explicitly toward public and media communication: one interdisciplinary initiative paired veterinary and communication students to rehearse television interviews and public speaking [95], while risk communication has been embedded within public health and epidemiology coursework using a clinical framing to reach beyond it [67]. Interprofessional education bringing veterinary and human health students together is likewise emerging [32], and the articulation of Day One public health competencies signals an intent to treat One Health communication as core rather than optional [47]. What these examples share, however, is their character: most are pilots, electives or single-institution innovations rather than systematically embedded, sequenced and assessed strands, and the broader move to competency-based education is itself still unevenly implemented across institutions [96].

Consequently, the overall picture is one of real but unequally distributed strength. On the one hand, the strength of clinical communication teaching is real and should be preserved; the problem is not what is taught but what is left largely untaught around it. On the other hand, the promising initiatives that do reach beyond the dyad tend to be local, elective or pilot in character rather than systematically embedded and assessed across a program. The consequence is a graduate well prepared for the consulting room but variably prepared for the interprofessional, community, public health and digital demands that the expanded professional role now places upon them. Addressing this does not require displacing the clinical core; it requires extending the same competency-based logic outward along the continuum and specifying how the resulting competencies are to be structured, sequenced and assessed. What gets assessed gets valued. This is essential if you want communication to be genuinely valued in veterinary curricula. One could add that communication should be assessed longitudinally across the curriculum rather than in a single communication module. This recommendation is strongly aligned with the recent veterinary literature, which calls for systematic, longitudinal integration and assessment. Also, within the United Kingdom Continuous Professional Development (CPD) guidelines for seasoned veterinarians, communication is referred to as essential, grounding principles and advising for mechanisms and themes to be advanced in favor of trusting vet-client/community relationships [97].

## 7. An Integrated Communication Competency Continuum

**Figure 1:**
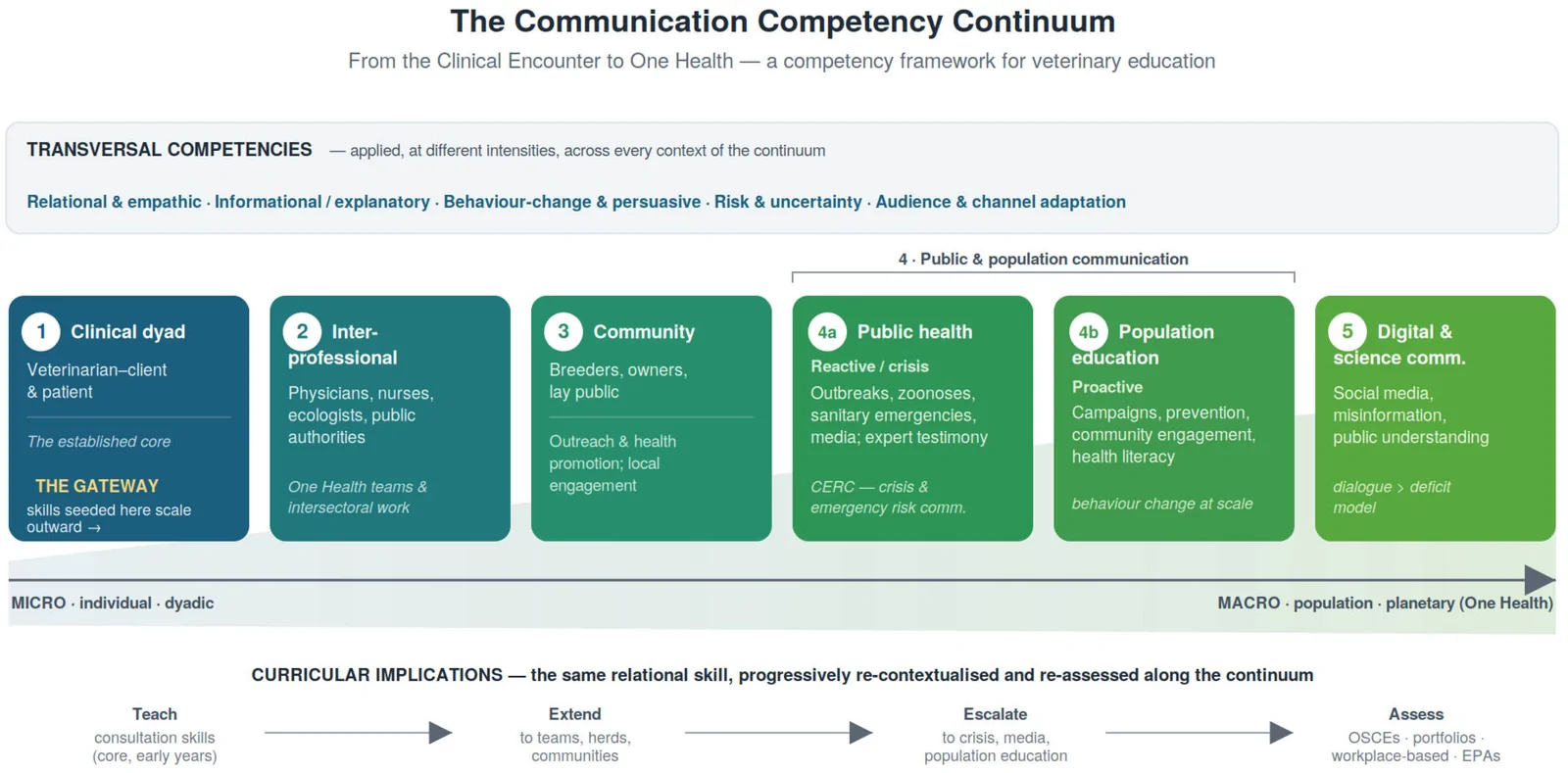
The Communication Competency Continuum for veterinary education. Communicative contexts are ordered by widening scope, from (1) the clinical dyad (the veterinarian–client consultation, the “gateway”), through (2) interprofessional, (3) community and (4) public and population communication (reactive/crisis, 4a; proactive/educational, 4b), to (5) digital and science communication, while five transversal competencies (relational and empathic; informational/explanatory; behavior change and persuasive; risk and uncertainty; audience and channel adaptation) recur across them. The lower band summarizes the main curricular implications (teach, extend, escalate, assess). The continuum is anchored in established competency frameworks: Competency-Based Veterinary Education [4]; the EAEVE’s Day One Competences [44]; the CanMEDS Communicator role [45]; the One Health Core Competencies [46]; and, at the clinical core, the Calgary–Cambridge communication guide [2]. Abbreviations: CERC, crisis and emergency risk communication; EPA, entrustable professional activity; OSCE, objective structured clinical examination.

The previous sections describe a profession whose communicative demands extend well beyond the consultation, and a curriculum whose strengths remain concentrated within it. This section sets out a constructive response: an integrated Communication Competency Continuum that keeps the clinical dyad as its foundation while extending the same competency-based logic outward to the One Health mandate (Figure 1).

### 7.1. The Continuum and Its Transversal Competencies

The framework is organized along two axes. The horizontal axis is a continuum of communicative contexts, ordered by scope from the individual to the population: the clinical dyad; interprofessional and intersectoral collaboration; community engagement; public and population communication, in both the reactive/crisis and proactive/educational modes; and the digital and science communication sphere that cuts across them all. The clinical dyad is positioned not as one context among equals but as the gateway, i.e., the site where communicative competence is first built and from which it is progressively re-contextualized. The vertical axis is a set of five transversal competencies that recur, at different intensities, across every context: relational and empathic communication; informational and explanatory communication; behavior change and persuasive communication; risk and uncertainty communication; and audience, register and channel adaptation. The organizing claim of the framework is that these competencies are continuous, not discrete. The relational skill that builds rapport with an anxious owner underlies trust building with a farming community; the explanatory skill that renders a diagnosis intelligible underlies public explanation of a zoonotic risk; the capacity to support a single client’s behavior change underlies population-level behavior change; and the ability to adapt to one interlocutor underlies fluency across registers, media and platforms. What changes along the continuum is not the underlying competency but its scale, its audience and the stakes and constraints attached to it. This is what distinguishes a continuum from a checklist of separate skills, and it is what makes the extension teachable: each new context builds on a competency the graduate already possesses.

It should be stressed that this ordering is a pedagogical and organizational device, not a claim about how communication unfolds in practice. In real professional activity, these contexts and competencies frequently coexist and interact. A single consultation may be dyadic, interprofessional and public facing at the same time. The continuum therefore supports progressive and iterative learning and curricular sequencing, rather than imposing a strict hierarchy or a temporal order on veterinary communication.

### 7.2. Anchoring and Graduating the Competencies

The framework is deliberately additive: it threads communication through existing competency architectures rather than proposing a competing one. At the dyadic core, it aligns with the communication competency domain of competency-based veterinary education and its associated entrustable professional activities [4,43,90,97], with the European Day One Competences [44], and with the “Communicator” role articulated in CanMEDS [45]. Its boundaries align with frameworks already focused on the wider role: the interprofessional context onto the collaborative competencies of interprofessional education [69]; and the public health, community and One Health contexts onto articulated One Health core competencies [46] and the Day One public health competencies defined for the veterinary workforce [47]. The continuum’s contribution is not to invent new competencies but to connect these dispersed strands into a single developmental line, making explicit that they are contexts of one communicative capability rather than unrelated requirements.

These same frameworks also supply the logic for graduating expectations along the continuum. Extending communication across its full breadth need not mean expecting every graduate to master crisis broadcasting or national campaign design. The framework therefore distinguishes a foundational literacy across all contexts, expected of every graduate as a Day One competency—the ability to communicate responsibly with colleagues across disciplines, with communities, and in the digital public sphere—from advanced competencies in crisis and media communication, risk broadcasting and population-campaign design, appropriate to those entering public health, production or research pathways [47]. The timing for such an articulation is opportune. The World Organisation for Animal Health (WOAH) has convened an ad hoc group to revise its Recommendations on the Competencies of Graduating Veterinarians (“Day 1 Graduates”) and the accompanying Veterinary Education Core Curriculum, both produced more than a decade ago, with the stated aim of aligning minimum standards of veterinary education with current scientific knowledge and recognized societal needs [98]. Notably, the call for experts to undertake this revision requires the ability to integrate emerging trends, such as technological advances and One Health challenges, into forward-looking educational frameworks, and lists among the expected transversal skills respectful communication, dialogue facilitation and consensus building [99]. These are precisely the capabilities the continuum seeks to cultivate in graduates. We argue that the revised guidelines should make them explicit at Day One level, framing communication not as a discrete clinical skill but as a capability that extends from the consultation room to the interprofessional, public health and digital spheres.

This graduated expectation keeps the proposal proportionate rather than maximalist, and mirrors how the profession already treats competencies such as surgery or diagnostic imaging, where routine proficiency is expected of every graduate and advanced proficiency is reserved for specialist training.

### 7.3. Making It Assessable

A competency that cannot be assessed cannot be assured, and the credibility of the framework rests on its assessability. At the dyadic end, the instruments already exist and are mature, including objective structured clinical examinations and simulated-client encounters [17] and validated observational measures of consultation processes such as shared decision making [7]. The task is to extend assessment outward with the same seriousness, adapting established methods to each context: interprofessional simulations and workplace-based assessment for collaborative competence; community and service-learning placements with structured reflection for community communication [93]; simulated media encounters for public and science communication [95]; and portfolios and entrustable professional activities to capture performance longitudinally across the continuum [43]. Framing outcomes at the level of behavior and results, rather than reaction alone, would allow programs to demonstrate that competencies acquired in the classroom transfer to practice. A useful diagnostic in curriculum review is to cross-tabulate context against assessment: where a context is taught but not assessed, the competency remains aspirational.

### 7.4. Curricular Implications

Operationalizing the continuum implies vertical, spiral integration rather than a bolt-on module: the same relational and explanatory competencies are introduced in the consulting room context in the early years, then deliberately extended to teams, groups and communities, escalated to public health, crisis and population settings, and assessed at each stage against context-appropriate standards. Such integration can build on evidence-based principles of communication-curriculum design [89] and, crucially, need not be invented from nothing: the isolated innovative pedagogies identified in an earlier section, such as the community-engagement and service-learning programs [92,93], production-medicine communication training [94], interdisciplinary media-communication exercises [95], risk-communication assignments [67] and interprofessional education [32], already provide tested templates for individual contexts. The framework’s role is to convert these scattered examples into a coherent, sequenced and assessed strand, attending to generational communication culture and implicit value. The practical constraints are real. They include the competition for curricular space and the uneven implementation of competency-based reform [96], together with the faculty development that teaching beyond the dyad requires. Shaw and Coe [100] advise for groundwork practices in veterinary education, referring to communication toolbox from appreciating communication to build and sustain relationships up to enhance clinical outcomes and team collaboration via structured teaching of consultations and iterative feedback.

## 8. Implications and a Research Agenda

Adopting the continuum has immediate implications for the three players that shape veterinary communication competence. For veterinary schools, the priority is integration rather than addition: the competencies of the wider contexts are best introduced by extending existing consultation teaching outward, reusing the tested examples identified before, from community-engagement and service-learning placements [92,93] to production-medicine communication [94], simulated media encounters [95] and interprofessional education [32], and sequencing them vertically against evidence-based principles of curriculum design [89]. For accreditors and standard-setters, the implication is to make communication beyond the dyad an explicit and assessed expectation, building on the Day One public health competencies already articulated for the workforce [47] and on the communication domain of competency-based frameworks, so that provision is driven by standards rather than left to local initiative. For continuing professional development providers, the continuum is a reminder that much of the demand falls on veterinarians already in practice: evidence that structured communication training improves the population-facing conversations of practicing food-animal veterinarians [94] suggests that Continuous Professional Development (CPD) is a realistic and underused route to competence across the wider contexts.

Realizing this vision, however, exposes how much remains unknown, and the framework is as much an agenda for research as a prescription for practice. The most fundamental gap concerns transfer. The continuum assumes that competencies seeded in the dyad can be re-contextualized outward, but the extent to which dyadic skill actually transfers to interprofessional, community and public settings, and how best to scaffold that transfer, has not yet been established empirically. A second gap concerns assessment beyond the dyad. Robust, validated instruments exist for the consultation, including observational measures of shared decision making [7] and mature simulated-client and OSCE methods [17], but equivalents for interprofessional collaboration, community engagement and public or media communication are largely lacking; developing and validating them is a precondition for treating these competencies as assured rather than aspirational. A third gap respects the outcomes: much of the existing educational literature reports learner reactions and short-term learning rather than proved changes in behavior or downstream results, and studies designed to capture higher-order outcomes are needed if the continuum’s claims are to be tested rather than assumed.

Two further priorities intersect with these. Faculty development is a practical bottleneck. Teaching credibly beyond the dyad requires educators comfortable with risk communication, interprofessional facilitation and science communication, and the workforce to deliver this is itself thin. Thus, who teaches the teachers remains an open question. And the framework’s cross-cultural and international applicability cannot be assumed: curricular structures, regulatory roles and public communication cultures differ across jurisdictions, and a continuum developed largely from Anglophone and European evidence will need testing and adaptation elsewhere. The digital and science communication context is a particularly moving target, in which even the evidence base on countering misinformation is unsettled [39] and veterinary-specific study remains scarce. A related and largely unexplored resource is the intersectionality framework [101], which examines how individuals’ overlapping social identities, and the structural inequalities attached to them, shape how messages are received and how trust is established. Applied to veterinary communication, it offers a principled basis for teaching audience-adaptive, culturally responsive communication with diverse clients, communities and publics, and it is especially pertinent to interprofessional and One Health settings, where practitioners must reach populations, whose circumstances differ widely. Its near absence from veterinary communication curricula is itself a gap worth addressing. This article is framed around One Health; a complementary One Welfare lens is equally pertinent. Veterinarians’ own handling of animals is itself a form of (non-verbal) communication and a model for clients, so promoting low-stress, fear-free and cooperative-care approaches, and improving practitioners’ ability to read species-specific animal behavior, their expressions and other forms of communication, is also part of the profession’s communicative role. Extending the continuum to encompass animal–human communication and to ensure One Welfare in practice is a worthwhile direction for future development [102,103].

However, none of this diminishes the case for acting now. The constraints are real. Although competition for curricular space and the uneven implementation of competency-based reform [96] are highly placed among them, they argue for incremental, shared and well-evaluated integration rather than for deferral. The continuum offers a structure within which individual innovations, currently scattered and unassessed, can be connected, sequenced and studied, so that the profession’s communication education comes to match the breadth of the role it now serves.

## 9. Conclusions

Communication has become an accepted clinical competency in veterinary medicine, taught and increasingly assessed at the level of the consultation. This paper has argued that the consulting room, for all the sophistication of the teaching built around it, is the beginning of veterinary communication rather than its limits. The same encounter in which a clinician counsels a single client is also the profession’s most frequent point of contact with antimicrobial stewardship, zoonotic risk, food safety and public trust in science; the clinical dyad is, in this sense, a gateway to communication that reaches interprofessional teams, communities, populations and the digital public sphere.

Reading these contexts as a continuum rather than as a list of separate demands has a practical payoff. It shows that the relational, explanatory, persuasive and risk-oriented competencies a graduate first builds with an anxious owner are the very competencies, re-contextualized, and with new stakes attached, that the wider One Health role requires, and it makes their extension teachable and assessable rather than left to chance. The integrated Communication Competency Continuum proposed here is offered in that spirit: not as a replacement for clinical communication training, which should be preserved, but as a way of connecting the profession’s scattered educational innovations into a single, sequenced and anchored developmental line.

Educating veterinarians for the role they actually occupy means teaching them to communicate across the whole of it. The consultation remains the foundation; the task ahead is to build outward from it, deliberately, until the reach of veterinary communication education matches the reach of veterinary responsibility.

## Data Availability

The original contributions presented in this study are included in the article.
